# A qualitative study on the vulnerability factors of unintentional injuries among children aged 3–12 in Guizhou Province, China, based on the social-ecological model

**DOI:** 10.3389/fpubh.2026.1853061

**Published:** 2026-07-27

**Authors:** Xiujuan Li, Roumei Lin, Liping Li, Pan Wen, Linlin Xie, Yaogui Lu, Zicheng Cao, Fancun Meng, Yanhong Huang, Jian-E Peng

**Affiliations:** 1 School of Public Health, Shantou University, Shantou, China; 2 Injury Prevention Research Center, Shantou University Medical College, Shantou, China; 3 Mental Health Center of Shantou University, Shantou, China; 4 College of Liberal Arts, Shantou University, Shantou, China

**Keywords:** children aged 3–12, unintentional injuries, vulnerability factors, qualitative research, interviews

## Abstract

**Background:**

Unintentional injuries represent a primary cause of child mortality worldwide. However, child injury research in China exhibits a distinct disparity in research coverage; while studies on urban populations are abundant, deep qualitative research exploring multi-level vulnerability factors among children in rural southwestern regions remains scarce.

**Objective:**

This study aimed to explore and conceptualize the multi-level vulnerability factors for unintentional injuries among children aged 3–12 years in Guizhou Province.

**Methods:**

Semi-structured interviews were conducted with 290 participants, including children, their caregivers, teachers, and local social workers across three regions in Guizhou. Data collection involved focus group discussions with children and in-depth interviews with adults. We conducted thematic analysis using NVivo 15 software.

**Results:**

Data analysis yielded four overarching themes encompassing 16 sub-themes: (1) Family vulnerability factors (absence of guardianship, inadequate parental safety initiative, emotional alienation, hazards in the home environment, and economic constraints); (2) Children’s individual vulnerability factors (weak safety awareness, risks of daily activities in the absence of supervision, personality traits, and disabilities); (3) Community vulnerability factors (safety hazards in the community environment and facilities, inadequate safety promotion, Insufficient interdepartmental collaboration, insufficient social attention, and imbalance of power and responsibility); (4) School vulnerability factors (safety hazards in the school environment and facilities, and inadequate safety education). Among these, the absence of family supervision, inadequate parental safety initiative, and home environmental hazards exacerbated by economic pressure emerged as core vulnerability factors. Furthermore, this study identified the cultural phenomenon of “filial silence,” reflecting how traditional filial piety influences injury concealment among children.

**Conclusion:**

Unintentional childhood injuries in Guizhou stem from intersecting multi-level vulnerabilities. The phenomenon of “filial silence” presents unique cultural challenges for injury surveillance. Interventions must integrate immediate environmental modifications, collaborative guardianship networks, and long-term rural revitalization strategies.

## 1 Introduction

Unintentional injuries among children are defined as external, sudden, and unintended events that cause harm to their health and life (1). The International Classification of Diseases, 10th Revision (ICD-10), categorizes unintentional injuries into 10 types based on external causes of morbidity and mortality, including falls, road traffic injuries, drowning, and others (2). According to World Health Organization (WHO) data, in 2016 alone, injuries and violence caused over 640,000 deaths among children aged 0–14 years globally, representing the leading cause of mortality in this age group, with unintentional injuries accounting for more than 90% of these fatalities (3). The Chinese Death Cause Surveillance Dataset (2021) reports an injury mortality rate of 8.86 per 100,000 among children aged 1–14 years, with unintentional injuries similarly accounting for more than 90% of these fatalities (4). Furthermore, children aged 3–12 years are particularly vulnerable to unintentional injuries because of rapidly developing motor skills and limited risk-recognition abilities (4, 5).

Unintentional injuries among children have serious consequences, ranging from mortality to trauma and disability, and thus impose significant burdens on individuals and society (6). Existing evidence indicates that these injuries are preventable, with their risk significantly influenced by individual traits and social environmental factors (7).

Current research on children’s unintentional injuries predominantly focuses on the description of epidemiological characteristics and individual-level cognitive behavioral analysis (8, 9). Moreover, most of the research samples are from educational institutions in urban areas (10), while in-depth investigation on vulnerability factors in rural western regions remains scarce. Vulnerability, defined as the susceptibility of individuals or groups to suffer harm when confronted with risks, is shaped by factors at the individual, familial, communal, and societal levels (11). In rural southwestern China, child injury risks exhibit intricate regional characteristics driven by structural poverty, labor migration, and traditional cultural beliefs (12). For example, Guizhou Province, a prominent labor-exporting region, harbors a substantial number of left-behind children, constituting nearly half of the rural child demographic (13). These children encounter heightened injury risks and more severe consequences due to the absence of guardianship, restricted access to healthcare, and deficiencies in educational resources. Additionally, children with disabilities, who experience higher injury rates due to disabilities and unsuitable environments, have been largely overlooked in mainstream research (14). These vulnerable populations urgently require targeted interventions.

In recent years, the Social-Ecological Model (SEM) has gained prominence in injury research (15). This model emphasizes multi-level interactions among individuals, families, communities, and societal environments, offering a comprehensive theoretical framework for injury prevention. Existing research predominantly focuses on urban childhood injuries (16, 17), while studies on unintentional injuries among children in rural western China remain scarce. To address this gap, this study employs the SEM as its theoretical framework and utilizes qualitative research methods to explore vulnerability factors associated with unintentional injuries among children aged 3–12 years in Guizhou Province. By analyzing interactions across four dimensions—child, family, school, and community, we aim to identify critical vulnerability factors and propose targeted prevention strategies and policy recommendations. The findings will provide scientific evidence for child safety governance within the Chinese rural revitalization context and serve as a valuable reference for injury prevention practices in similar developing-country settings.

## 2 Methods

### 2.1 Study design

This qualitative study was conducted from July 8–22, 2024, utilizing semi-structured interviews to explore children’s unintentional injuries through participants’ experiences and perspectives. Interviews included focus group interviews with 169 children (5–6 per group) and in-depth interviews with 121 adults (caregivers, teachers, and local social workers). All interviews were conducted face-to-face in Mandarin, audio-recorded, and lasted 30–45 min.

For the subgroup of younger children (aged 3–5 years), the data collection process was operationally adapted to accommodate their developmental stage. Recognizing that young children cannot conceptualize or evaluate abstract vulnerability factors, the semi-structured focus groups utilized highly concrete, situational prompts (e.g., asking how they interact with specific household objects like kettles, rather than asking about general safety risks). The children’s narratives and behavioral responses served purely as raw observational data. If a child did not answer in accordance with the prompt or became distracted, the facilitator gently redirected their attention using picture cards or toys. Crucially, off-topic responses or silences were not forced but were documented as potential indicators of insufficient risk perception, ensuring that complex hazard evaluation was not inappropriately forced upon them.

The study strictly adhered to the Declaration of Helsinki ethical guidelines, and all participants signed informed consent before the interview. The interview process was conducted according to the interview guides specially designed for this study (Please see Supplementary File 1 for the detailed interview guides), and the results were reported in accordance with the Consolidated Criteria for Reporting Qualitative Research (COREQ) guidelines (18). Ethical approval was granted by the Shantou University Ethics Committee (approval number: STU2024006001).

### 2.2 Recruitment and participants

We employed purposive sampling to recruit participants from Central Guizhou (Guiyang City), Southeast Guizhou (Liping County), and Southwest Guizhou (Xingyi City). Participants included children aged 3–12 years, their caregivers, teachers, school administrators, and local social workers. The research team collaborated with local social work organizations and invited volunteers to participate in the interview via WeChat and telephone. Focus group interviews were conducted with children, and in-depth interviews were conducted with adults. All interviews were conducted at social service agencies. The study adhered to informed consent principles: child participants required informed consent from their guardians or proxies, and all participants were informed that their participation was voluntary and that their information would be kept confidential. Inclusion criteria were (1) children aged 3–12 years and their primary caregivers and (2) those who signed the informed consent. Exclusion criteria were (1) children outside the 3–12 age range and their caregivers and (2) guardians who refused to participate or did not sign the informed consent.

While the overall sample size (*n* = 290) is relatively large for a qualitative study, this was methodologically necessitated by our SEM framework, which requires capturing multi-level perspectives. To ensure robust thematic depth and achieve data saturation within each sub-system, it was essential to purposefully sample across three diverse geographic regions, three distinct child sub-populations (left-behind, non-left-behind, and disabled), and four adult stakeholder groups (caregivers, teachers, administrators, and social workers).

### 2.3 Data analysis

We conducted thematic analysis utilizing a hybrid deductive-inductive approach guided by the constant comparative method (19). Deductively, the Social-Ecological Model (SEM) served as the overarching analytical framework to structure the data into macro-levels of vulnerability (individual, family, community, and school); inductively, open coding was applied to capture nuanced, culturally specific sub-themes directly from the narratives. Interviews were transcribed verbatim, and data collection and analysis were carried out simultaneously to iteratively refine subsequent interviews. Thematic saturation was confirmed by the data saturation principle (20). Data saturation was dynamically monitored and operationalized at the sub-group level; participant recruitment for a specific demographic was ceased only when consecutive subsequent transcripts yielded no new codes or theoretical insights within the established SEM framework.

Coding proceeded in two phases. In phase 1, two researchers (Li and Wen) independently performed open coding on five transcripts using NVivo 15 software. In phase 2, three researchers (Li, Wen, and Lin) collaboratively coded another transcript to ensure coding consistency. We refined the analytical framework through team meetings, discussing coding results and memos, removing insufficiently evidenced themes, and merging similar ones. Subsequently, researcher Li completed the coding of the remaining transcripts. Following two theme-review meetings, we invited two independent researchers (Meng and Cao) to validate the final analytical framework. Core themes were determined through discussion among four researchers (Li, Lin, Meng, and Cao). For the subgroup of younger children (aged 3–5 years), themes such as “weak safety awareness” and “risks of daily activities” were not self-identified by the children; rather, they were analytically derived by the researchers based on the children’s demonstrated lack of hazard recognition. To validate these findings, a community-level thematic data source triangulation approach was employed. The cognitive constraints and risky behaviors described by the children were systematically cross-referenced and contextualized against the structural hazards and supervisory gaps detailed in the in-depth interviews with adult caregivers and teachers from the same socio-ecological settings. Data was anonymized, and each participant was assigned a unique identifier.

## 3 Results

### 3.1 Sample characteristics and distribution

**Figure 1:**
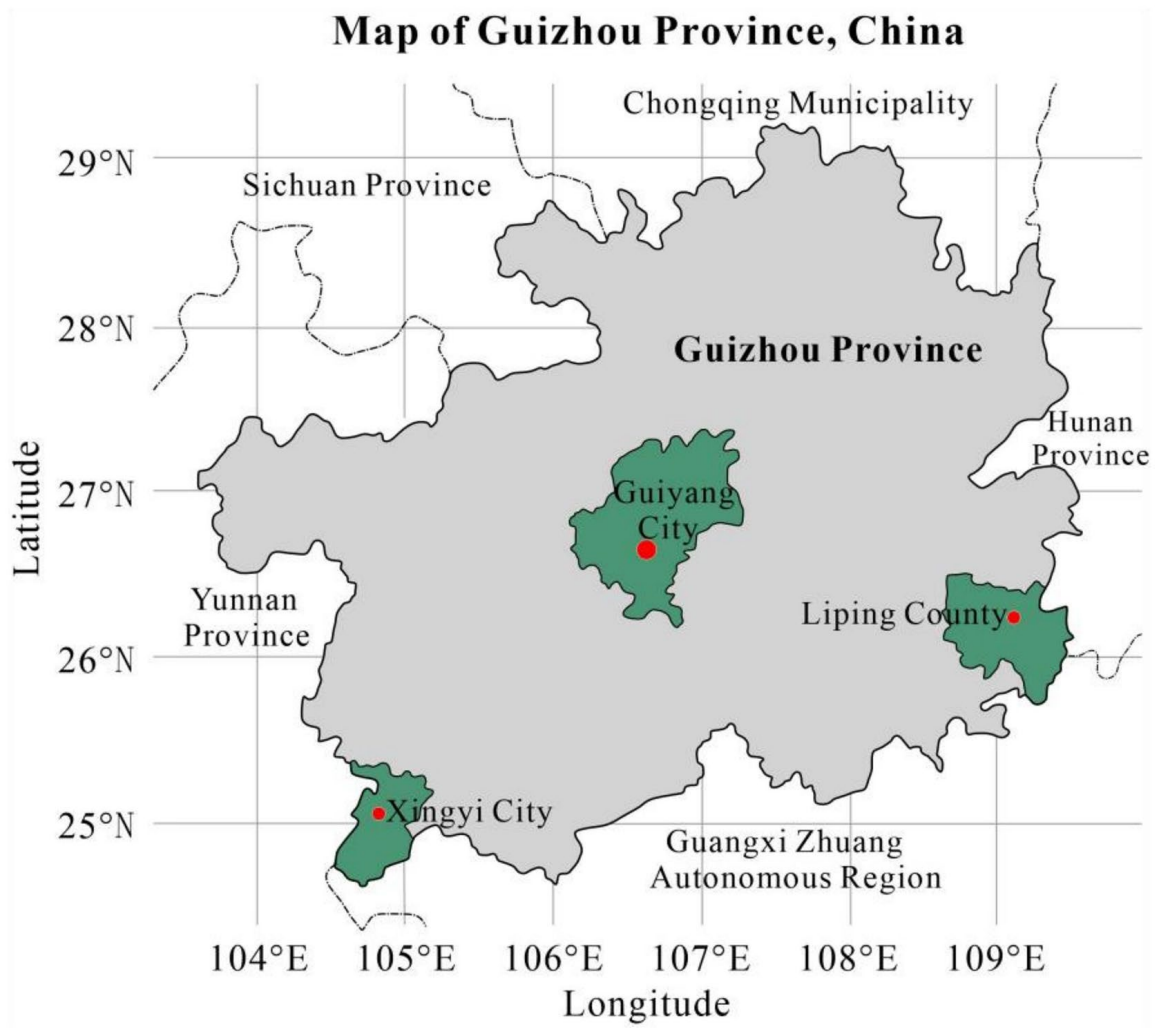
Geographic location of Guiyang City, Liping County and Xingyi City, Guizhou Province, China.

This research was conducted in Guiyang City, Liping County, and Xingyi City within Guizhou Province (Figure 1), resulting in 152 valid interviews (31 focus group interviews and 121 in-depth interviews) with a total of 290 participants. The in-depth interviews involved 121 adult participants, comprising 77 child caretakers, 22 teachers, 7 school administrators, and 15 social workers; the focus group interviews included 169 children (86 boys and 83 girls, among whom 14 were children with disabilities) (Table 1).

**Table 1:** Distribution of valid samples by age category.

Age group	Left-Behind children		Non-left-behind children		Children with disabilities		Caregivers of left-behind children			Caregivers of non-left-behind children			Caregivers of disabled children		
	Girl	Boy	Girl	Boy	Girl	Boy	Father	Mother	Grand-parents	Father	Mother	Grand-parents	Father	Mother	Grand-parents
3 ~ 5 years old	18	20	7	8	2	4	3	4	4	2	5	0	1	2	0
6 ~ 9 years old	21	30	5	1	5	3	1	8	9	0	4	0	1	6	0
10 ~ 12 years old	23	16	2	4	0	0	2	3	7	1	5	0	3	5	1

The study sample included three distinct groups: left-behind children primarily from Liping and Xingyi, non-left-behind children centered in Guiyang, and children with disabilities, namely cerebral palsy and hearing impairment. During the data collection procedure, two interviews (4 people) were removed owing to recording quality difficulties, ensuring the reliability of the final data.

This study identified four primary themes and 16 sub-themes through a multi-perspective analysis: (1) Children’s individual vulnerability factors (267 codes); (2) Family vulnerability factors (491 codes); (3) School vulnerability factors (86 codes); and (4) Community vulnerability factors (199 codes). It should be noted that while coding frequencies are presented to illustrate the relative prominence and recurrence of themes within our purposive sample, they are interpreted cautiously as qualitative indicators of thematic weight, rather than statistical probabilities generalizable to a broader population (Table 2).

**Table 2:** Themes and sub-themes identified.

Themes	Sub-themes (Sample code)	Frequency of code
Children’s individual vulnerability factors	Weak safety awareness (insufficient risk perception; imitation of dangerous behaviors)	77
	Risks of daily activities in the absence of supervision (household chores; routine hazardous activities)	70
	Personality traits (bold; curious; and energetic)	65
	Disabilities (physical dysfunction; communication barriers)	55
Family vulnerability factors	Absence of guardianship (a lack of energy and ability; lax supervision; parental inattention; parental role deficiency)	250
	Inadequate parental safety initiative (insufficient safety awareness; inadequate safety education)	112
	Emotional alienation (fear-driven concealment; filial silence)	56
	Hazards in the home environment (structural deficiencies in buildings; improper management of dangerous items)	51
	Economic constraints (—)	22
School vulnerability factors	Safety hazards in the school environment and facilities (environmental hazards; inadequate medical facilities)	63
	Inadequate safety education (incomplete content coverage; single form; Insufficient home-school cooperation)	23
Community vulnerability factors	Safety hazards in the community environment and facilities (infrastructure hazards; environmental hazards)	82
	Inadequate safety promotion (incomplete content coverage; single form; and limited resources and capacity)	70
	Insufficient interdepartmental collaboration (—)	17
	Insufficient social attention (—)	15
	Imbalance of power and responsibility (—)	15

### 3.2 Children’s individual vulnerability factors

Children’s individual vulnerability factors are the primary contributors to the occurrence of unintentional injuries among children. Four sub-themes emerged from the interview data.

#### 3.2.1 Weak safety awareness

Two main aspects of children’s weak safety awareness are their insufficient risk perception and their Imitation of dangerous behaviors. At the cognitive level, most children lack the ability to recognize risks and are not vigilant regarding hazardous objects. For example, a left-behind boy was burned and marked by engaging with his cell phone while disregarding a basin of boiling water situated on a bench [#120 left-behind children]. This cognitive constraint stems directly from insufficient safety teaching at home and in educational institutions, as articulated by one educator:

*“If parents and teachers do not explicitly convey the dangers associated with certain behaviors, children may engage in risky actions due to ignorance.” [#132 Teacher at institutions for the handicapped]*

At the behavioral level, children are susceptible to external inducements to imitate dangerous actions, such as jumping from heights in movies and television shows [#91 Social Worker Leadership]. These behavioral risks are further amplified by lack of supervision and environmental changes. For example, left-behind children migrating from rural to urban regions face an elevated risk of accidents due to their unfamiliarity with driving regulations [#145 Social Worker Leadership].

#### 3.2.2 Risks of daily activities in the absence of supervision

Children are often exposed to several safety hazards when undertaking household chores beyond their chronological age, as their emergency management skills may struggle to address potential risks. For instance, the 9-year-old girl was burned by hot oil cooking alone and then soaked in ice water [#96 left-behind children]—the scientific treatment should be continuous cold water rinsing rather than ice packs. The social worker interns also observed the situation of “children cooking alone when parents are drinking after work” in families where children are not left behind [#93 Social Worker].

In the absence of effective supervision, children are easily exposed to everyday hazardous activities, such as being blown up by playing with firecrackers or stung by poking at a beehive. Moreover, certain children display recurring damage patterns resulting from enduring inadequacies in risk perception and avoidance. For example, a 10-year-old boy was involved in five accidents within a year [#106 left-behind children].demonstrating a chronic deficiency in risk perception. Daily activities can be transformed into a source of serious injury when supervision deficits and children’s cognitive limitations are superimposed.

#### 3.2.3 Personality traits

Generally, most adolescents exhibit courage, curiosity, and vigor, which drives them to independently confront risks, and they are more inclined to identify hazards by personal experimentation rather than through lectures. The efficacy of safety instruction is diminished by constrained memory retention. Children face an elevated risk of accidental injuries owing to the interplay of these behavioral traits.

*“Children are curious about everything they see and will imitate adults operating induction cookers and kitchen knives.” [#51 mother of non-left-behind children]*

*“He cannot remember what he’s been taught about safety and forgets it when he’s finished.” [#10 mothers of left-behind children]*

#### 3.2.4 Disabilities

Individual children have disabilities that make them high-risk individuals for injury. Children with physical impairments in this study include those with cerebral palsy, autism, and hearing impairments, hereafter referred to as children with disabilities. Their safety risks mainly stem from two core factors: physical dysfunction directly increases the risk of accidents such as falls and choking. Communication barriers make it difficult to pass on safety knowledge, and even repeated education is not effective in changing risky behavior patterns. These physical constraints trap children with impairments in a never-ending cycle of damage, forcing them to rely completely on the protection of their guardians.

*“Most children with cerebral palsy are uncoordinated and walk with an unsteady center of gravity. Severely affected children with cerebral palsy cannot even sit still and are prone to falls or falls from heights. And their chewing and swallowing functions are very poor, making them prone to foreign objects getting stuck waiting.” [#140 Teachers at institutions for the handicapped]*

*“Hearing-impaired children walking down the road cannot recognize danger by sound, cannot avoid it in time, and are vulnerable to traffic injuries.” [#132 Teachers at institutions for the handicapped]*

*“Children with autism can run and jump but have their own ideas and play styles, and basically will not listen to safety instructions and cannot communicate.” [#140 Teachers at institutions for the handicapped]*

### 3.3 Family vulnerability factors

Family vulnerability factors are the principal risk source for unintentional injuries in children aged 3–12 years in Guizhou Province, categorized into six subthemes.

#### 3.3.1 Absence of guardianship

Absence of guardianship refers to children being frequently exposed to the danger of unintentional injury as a result of parents’ or primary guardians’ failure to adequately carry out their guardianship duties. This primarily encompasses four factors: a lack of energy and ability, lax supervision, parents’ inattention to the prevention and control of unintended injuries, and parental role deficiency.

First, a lack of energy and ability, coupled with parental role deficiency, severely limits supervision across vulnerable family structures. Financial constraints frequently force single parents to compromise supervision for survival: “After my husband’s death, I raised my two children on my own. Due to financial difficulties, I had to work part-time, leaving my children unsupervised on occasion” [#63 mother of non-left-behind children]. Similarly, extreme caregiving demands overwhelm families with disabled or multiple children. Guardians of children with cerebral palsy are “increasingly unable to hold their children as they grow older, and are unable to protect their children in time when they fall, often resulting in bruises” [#87 social worker], while multi-child families are “prone to child falls or bumps” due to the dispersal of energy [#42 mother of left-behind children]. This supervisory gap reaches its most critical level—parental role deficiency—among left-behind children living entirely alone. Without any parental presence, they often engage in highly dangerous behaviors, such as “touching 220-volt household electrical circuits and 1,000-volt high-voltage wires without authorization” [#129 School Leadership].

Second, lax supervision and parental inattention further exacerbate these risks even when caregivers are present. Age-related cognitive limitations often cause grandparent guardians to “adopt a model of free-range parenting, believing that children’s bumps and bruises are a normal part of growing up” [#92 social worker]. Conversely, younger parents often exhibit lax supervision through situational distraction, such as “looking at their cell phones while crossing the street with their children” [#77 mother of hearing-impaired children]. Furthermore, a pervasive inattention to injury prevention manifests in both proactive and reactive negligence. Caregivers frequently ignore evident home hazards—such as having “knives and medicines lying around the house” or letting “children cook their own food” [#93 social worker]—and often fail to implement crucial remedial procedures, such as seeking immunizations after dog bites [#111 left-behind children].

#### 3.3.2 Inadequate parental safety initiative

The absence of parental safety initiatives is primarily evident in two areas: insufficient safety awareness and inadequate safety education.

Insufficient safety awareness manifests across three key areas: inadequate risk perception, neglect of home hazards, and improper post-injury management. Initially, inadequate risk perception is evident, as some parents choose to allow their children to swim in natural lakes, believing that “there are fewer people in the lakes, the water temperature is cooler, and the water quality is cleaner” [#74 father of non-left-behind children], while overlooking the dangers associated with open water. Secondly, they turn a blind eye to the hidden dangers of home safety. A survey conducted among social workers revealed that numerous families store knives and medications haphazardly, in addition to keeping deep buckets without lids in bathrooms, among other unsafe practices [#85 social worker]. Thirdly, families often resort to unscientific folk remedies rather than seeking medical treatment post-injury, such as utilizing tomatoes to treat bee stings [#111 left-behind children] or applying tea oil for burns [#106 left-behind children].

The inadequacy of safety education is apparent in multiple facets. Due to their own limited knowledge, parents often provide overly generalized warnings, merely reminding children to “pay attention to safety,” without offering specific risk mitigation strategies [#11 father of non-left-behind children]. Furthermore, certain parents have an obvious tendency to transfer the responsibility for education, entrusting safety education entirely to educational institutions. Caregivers frequently justify this reliance by stating that “not much is taught at home, and teachers teach more” [#58 mother of children with cerebral palsy] or claiming that parents’ words “are not as effective as teachers” [#55 father of hearing-impaired children]. Finally, intergenerational custody arrangements often introduce severe communication barriers; for instance, some grandparents only speak local dialects like the Dong language, which fundamentally hinders their ability to provide effective safety guidance to their grandchildren [#132 Teacher at institutions for the handicapped; #95 left-behind children].

#### 3.3.3 Emotional alienation

Emotional detachment denotes a psychological condition in which children are reluctant or fearful to seek assistance from their parents following injury, stemming from a prolonged absence of emotional communication between parents and children. This study identifies two distinct psychological reasons for children’s concealment of injuries. First, fear-driven concealment, which is a passive and reactive behavior that occurs due to the fear of punishment. For example, Participant #119 (a non-left-behind child), having been previously blamed by their parents for their arms being crushed by bicycles, and then did not dare to report subsequent injuries. Second, the study identified the phenomenon of “filial silence.” Unlike fear-driven concealment, filial silence is an active and empathetic choice. It occurs when children deliberately hide their physical pain and injuries to avoid adding emotional or financial burdens to their already struggling parents. A left-behind child said, “My parents work so hard far away from home to earn money for my schooling. Usually, if I get injured, I will not tell them because they would worry and cry over the phone. Once, I got a deep cut on my hand, and I just wrapped it by myself.” Specific parenting styles may exacerbate this form of alienation; for instance, overemphasizing independence may cause children to choose to remain silent when they need help [#39 father of left-behind children]. This alienation not only postpones the prompt treatment of injuries but also significantly obstructs opportunities for family safety education.

#### 3.3.4 Hazards in the home environment

Household hazards primarily categorize into two types: improper management of dangerous items and structural deficiencies in buildings. Risks associated with dangerous items include uncovered deep water containers, exposed heating stoves, improperly installed electrical wiring, and haphazardly stored medications or chemicals.

*“In response to water outages in their communities, many families will use deep, uncovered buckets to store water and lack basic drowning prevention measures.” [#145 social worker]*

*“Many families place items such as oral medications, laundry detergents, and dishwashing liquids casually within a child’s reach, increasing the risk of accidental poisoning in young children.” [#145 social worker]*

Structural deficiencies are most prominent in the safety hazards of staircases, including wooden staircases without handrails [#145 social worker],straight staircases with steep, unguarded rungs [#71 mother of non-left-behind children],and high steps in bathrooms [#89 social worker]. All of these factors directly raise the risk of unintentional injuries to children.

#### 3.3.5 Economic constraints

Economic constraints serve as a foundational vulnerability that systematically amplifies both physical environmental hazards and supervision deficits. First, financial limitations force disadvantaged families into substandard and inherently hazardous living conditions. For instance, rural households frequently rely on “honeycomb coal for cooking and heating,” which significantly elevates the risks of “burns or carbon dioxide poisoning” [#93Social Worker]. Similarly, low-income urban families often reside in older buildings without elevators, where structural hazards like steep stairs pose severe fall risks, particularly for children with physical disabilities [#91 Social Worker Leadership]. Second, severe economic pressure directly depletes families’ capacity to invest in safe mobility and supervision. Driven by absolute survival needs, parents must prioritize labor over guardianship, as poignantly expressed by one mother, “I cannot take care of the kids if I go to work” [#38 mother of left-behind children]. Furthermore, this financial strain compromises safe commuting for vulnerable populations. “Unable to afford transportation, numerous children must traverse considerable distances to and from school unaccompanied, while the roads are congested with vehicles, increasing their susceptibility to traffic accidents” [#93 Social Worker].

### 3.4 School vulnerability factors

As the primary setting for children’s daily activities, environmental safety hazards and management negligence in schools may result in unintentional injuries. Two subthemes were identified from the interview data, presented in Table 2.

#### 3.4.1 Safety hazards in the school environment and facilities

The school environment presents multiple safety hazards, with inadequate anti-slip measures in public areas being a primary cause of student falls. Many children interviewed reported that they “fell due to slippery floors in the school cafeteria and restrooms” [#112 left-behind children]. Flaws in stairway design, combined with students’ risky behaviors, contribute to fall accidents. For example, narrow stairwells coupled with students chasing each other increase the likelihood of falls and trampling incidents [#109 left-behind children]. Inadequate protective facilities, e.g., “insufficiently high corridor guardrails and the absence of protective nets, put students at risk of falling from heights” [#143 School Leadership; #130 School Leadership]. Inadequate safety precautions in areas where younger children are active lead to a high incidence of burns and entrapment [#124school teacher], among others. Additionally, the absence of basic medical facilities and specialized personnel hinders the proper and timely treatment of injuries, often leading to delays in care. For example:

*“We do not have an infirmary at our school, nor is there a doctor specializing in wound treatment. After I hurt my leg in my last fall, the school could only call my mom to take me to the hospital.” [#109 left-behind children]*

#### 3.4.2 Inadequate safety education

School-based safety education is critically hampered by pedagogical rigidity and a systemic neglect of special educational needs. Primarily, safety instruction suffers from a superficial, one-size-fits-all approach lacking interactive engagement: “Our teacher primarily delivers safety knowledge through oral presentations during class meetings, lacking engaging formats such as videos and case studies” [#120 left-behind children]. The curriculum content is equally constrained and lacks contextual specificity to the children’s actual environments: “Our school mainly focuses on traffic safety, falls, and drowning prevention, with little attention given to other types of injuries. The promotional materials used are mostly downloaded from the internet, resulting in superficial content that lacks specificity” [#139 School Leadership].

Furthermore, the most vulnerable student populations face a complete absence of tailored safety curricula due to a structural professional deficit among educators. Educational institutions for children with disabilities operate with a critical gap in instructional capacity: “Our school primarily focuses on rehabilitation training and rarely teaches safety knowledge. Additionally, the teachers lack sufficient training in this area and do not fully understand it themselves” [#132 Teacher at institutions for the handicapped].

### 3.5 Community vulnerability factors

The community environment is a significant source of risk for unintentional injury to children. Five sub-themes were distilled from the interview data.

#### 3.5.1 Safety hazards in the community environment and facilities

The community environment exacerbates child injury risks through a critical combination of chronic infrastructural deficits and public management negligence. First, unregulated built environments and deficient infrastructure directly expose children to severe physical threats across both rural and urban settings. Structural hazards range from rural households featuring “a deep well… with no fence around it” [#116 left-behind child], to flawed urban infrastructure where underpass stairs are “highly inclined and sloping,” posing severe fall risks for the older adults and children [#74 father of non-left-behind children]. Furthermore, historical infrastructural debt in “older neighborhoods or isolated communities” often means there are “basically no firefighting facilities” [#133 Teacher], leaving vulnerable populations defenseless during emergencies.

Second, persistent gaps in public space and ecological management create unmonitored hazard zones. The absence of routine environmental oversight allows ecological threats to persist near educational institutions, where unsupervised students might “sneak out after school to pick fungi and play in the reservoirs,” risking both wild mushroom poisoning and drowning [#144 School Leadership]. Similarly, failures in basic public health governance are evident in the widespread lack of stray animal control. Residents frequently face risks from stray dogs and subsequent dog bites, exacerbated by a systemic lack of clear reporting channels to address these daily community hazards [#71 mother of non-left-behind children].

#### 3.5.2 Inadequate safety promotion

Community safety promotion suffers from narrow content coverage and severe resource deficits. Educational campaigns frequently focus on high-visibility risks while neglecting highly prevalent daily hazards and vulnerable populations: “Our community primarily focuses on promoting awareness of three types of unintentional injuries in children: drowning, traffic injuries, and falls, while other incidents are largely overlooked. However, dog bites and bee stings also occur frequently. Unfortunately, due to insufficient manpower, our outreach efforts remain incomplete and lack impact” [#145 Social Worker Leadership]. Similarly, parents lacking basic skills and those with disabled children are left without professional guidance: “I believe that public education on fire safety should be strengthened, as I lack the skills to operate a fire extinguisher” [#23 mother of non-left-behind children]. “There is a lack of safety education for children with disabilities in the community. We have a strong need for knowledge in this area but can only rely on online resources and shared experiences to learn independently” [#55 father of hearing-impaired children].

These superficial campaigns are fundamentally constrained by systemic resource shortages and a lack of authoritative backing. Grassroots organizations face acute operational shortages: “The intensity of publicity is undoubtedly insufficient. For example, due to limited human resources and funding, our community group can execute home visit campaigns only sporadically” [#88 social worker in disability institutions]. Practitioners emphasize that government leadership is urgently needed to bridge this credibility and capacity gap: “The government should enhance the promotion of knowledge regarding the prevention of accidental injuries to children, as its credibility ensures more effective outreach. Relying solely on social workers for this task is insufficient” [#81 social worker in disability institutions].

#### 3.5.3 Insufficient interdepartmental collaboration

Currently, there is a lack of effective collaboration among families, communities, schools, and government departments, resulting in unclear boundaries of responsibility for each subject. This leads to issues such as unaddressed hazardous areas outside schools and inadequate traffic management at school entrances. Additionally, some parents do not cooperate with safety education efforts. Even when schools attempt to engage parents for safety training through village leaders, the absence of a stable collaboration mechanism makes sustained cooperation difficult [#143 School Leadership].

*“Insufficient coordination among departments is a factor contributing to unintentional injuries in children. For instance, we discovered that the reservoir behind the school lacks guardrails and warning signs in hazardous areas. However, because it is outside our jurisdiction, we have no authority to address the issue and can only report it to the local government. Unfortunately, during this time, a drowning incident occurred, which is deeply distressing.” [#129 School Leadership]*

Additionally, social work organizations have limited resources and can only prioritize specific groups. Furthermore, “public participation in promotional activities is low due to the lack of appeal. There is an urgent need for the government to lead the establishment of a cross-departmental collaboration mechanism to integrate resources and jointly advance child safety initiatives” [#85 social worker in disability institutions].

*“The social work organizations are small in size and have insufficient manpower, so they can only temporarily help us supervise left-children living alone, but their level of cooperation remains minimal.” [#143 School Leadership]*

#### 3.5.4 Insufficient social attention

Currently, societal attention to child safety remains inadequate, characterized by a passive model of “neglect before incidents and concern afterward,” which makes it difficult to establish a long-term protective mechanism. In particular, the safety of special needs children has not received adequate attention, and public awareness of assistive devices for children with disabilities (e.g., hearing aids) is insufficient, making it difficult to form a social atmosphere of shared guardianship. In addition, “barrier-free facilities are virtually nonexistent, and problems such as the occupation of blind pathways and the lack of barrier-free access on flyovers are common” [#132 Teacher at institutions for the handicapped], further increasing the daily safety risks for children with special needs.

*“We often overlook unintentional injuries, taking them seriously only when an incident occurs, after which they are quickly forgotten.” [#80 social worker]*

#### 3.5.5 Imbalance of power and responsibility

The imbalance of power and responsibility refers to the structural asymmetry between administrative authority and protective accountability in preventing and controlling children’s unintentional injuries. Specifically, it manifests as upper-level authorities disproportionately shifting guardianship burdens onto schools, while government agencies with actual enforcement power engage in superficial, formalized safety supervision.

On the one hand, administrative mandates often force schools to absorb safety responsibilities that should be shared across society. For instance, “the education department, through measures such as shortening holidays, has overly concentrated the guardianship responsibility that should be shared among multiple parties within the school” [#145 Social Worker Leadership]. This results in schools bearing an excessive burden of guardianship while inadvertently undermining the primary role of parents.

On the other hand, government departments possessing the actual administrative power to eliminate environmental hazards often approach safety governance in a performative manner. For example, “when they visit grassroots levels to promote safety, they typically just take a few photos of their work and then leave, lacking effective supervision and failing to implement policies” [#81 social worker].

## 4 Discussion

This study, grounded in the Social-Ecological Model (15), identifies multi-level vulnerability factors contributing to unintentional injuries among children aged 3–12 years in Guizhou Province. The findings demonstrate that children’s injury risks arise from interactions across individual, family, school, and community layers.

At the individual level, children’s innate developmental and cognitive vulnerabilities serve as the primary baseline of risk. This baseline vulnerability is most devastating for children with disabilities, who encounter a compounding triad of challenges: disabilities, unsuitable environments, and limited social support. These factors underscore the inadequacy of current social welfare systems, including a scarcity of special education resources, a lack of accessible facilities, and a shortage of specialized caregivers. These deficits collectively hinder the safe development of children with disabilities, placing them at the margins of societal protection (21).

At the family level, family vulnerability factors act as the most immediate external environment interacting with these individual traits. The absence of guardianship must be understood within its broader structural context. This lack of supervision is not simply a manifestation of individual parental negligence, but a forced choice dictated by structural poverty and the household registration (Hukou) system (22). Driven by survival imperatives, rural parents are compelled to migrate for labor, while stringent institutional barriers prevent their children from migrating with them. It creates a “migrant parents-left-behind children” family separation pattern, forcing children into single-parent care, intergenerational guardianship, or even solitary living (23, 24). This phenomenon echoes the patterns of child injury caused by seasonal migration in rural India (25), suggesting that developing countries face common structural risks.

The finding of insufficient parental safety initiative challenges the explanatory boundaries of traditional health behavior theories. The study reveals that, despite acknowledging risks, some parents prioritize survival needs over safety investments due to economic constraints, creating a “cognition–behavior paradox” (26). For instance, impoverished families may be aware of the hazards of dilapidated wooden ladders but lack resources for renovation. Furthermore, economic strain exacerbates the home environmental hazards—such as living in older buildings without elevators and inadequate childproofing—which pose significant safety risks for economically disadvantaged families. This paradox demonstrates that cognitive safety education is fundamentally insufficient when divorced from socioeconomic realities; effective interventions must integrate concrete material assistance and environmental modifications to bridge the gap between risk awareness and preventive action.

This study identified the unique cultural phenomenon of “filial silence,” in which children proactively conceal their injuries to avoid burdening their parents. This contrasts with the passive concealment commonly observed in other injury studies, which is predominantly driven by fear of punishment or shame (27). Filial silence is an active, deeply empathetic choice. Grounded in the traditional Confucian ethic of filial piety and the pervasive cultural expectation for children to be sensible, children across various family structures internalize the need to prioritize family harmony. They acutely perceive the socioeconomic and emotional hardships of their burdened parents, whether due to labor migration, economic constraints, or daily life stresses. In response, they transform their own physical pain into a concealed moral sacrifice, hiding injuries to avoid exacerbating their parents’ stress. While reflecting profound family cohesion, this active concealment paradoxically creates a critical blind spot in injury surveillance and obstructs crucial opportunities for family safety education.

Furthermore, schools in China face a dilemma of responsibility-resource imbalance in child safety management. On the one hand, schools bear excessive management responsibilities. For example, the education department’s policy of shortening school vacations has disproportionately shifted safety management duties onto schools. On the other hand, schools encounter resource constraints, including insufficient school healthcare personnel and limited safety education methods. International evidence demonstrates that shared responsibility and complementary resource allocation are fundamental to sustainable safety governance (28). China urgently needs to achieve this transformation through specialized legislation and institutional innovation.

At the community level, multidimensional vulnerability characteristics are evident. In terms of environment, the open wells in rural areas and the fire hazards in old urban communities appear to be two separate environmental hazards on the surface, but they actually reflect the phenomenon of environmental inequality—safety resources are continuously tilted toward the advantaged groups, while the disadvantaged groups are forced to live in high-risk environments (29, 30). In terms of governance, insufficient resources and poor inter-departmental collaboration lead to inadequate safety promotion and management blind spots, for example, child drowning incidents occurring in administrative boundary waters. Simultaneously, the “campaign-style governance” approach (e.g., neglecting beforehand and paying attention afterward) coupled with the imbalance of rights and responsibilities further amplifies risks.

Based on these findings, we propose a multi-sectoral collaborative intervention mechanism with localized, tailored strategies: (1) at the family level, strengthen guardianship support networks. Furthermore, to dismantle the phenomenon of “filial silence,” caregivers should be trained in reassurance-based communication rather than punitive scolding, actively encouraging children to report injuries without fearing they are burdening their parents; (2) at the school level, innovate safety education approaches and improve emergency response systems, such as integrating pediatric first-aid training for school teachers to offset health personnel deficits and utilizing non-rigid, game-based safety curricula to accommodate children’s needs. (3) at the community level, implement a comprehensive “4E” governance framework: Engineering (prioritizing immediate funding for high-risk physical hazards identified in our study, such as fencing wild reservoirs in administrative boundary waters and capping open rural wells), Education (conduct stratified safety education), Enforcement (establish cross-departmental joint enforcement mechanisms), and Empowerment (cultivate community-based prevention capacity); Notably, child safety should be integrated into the rural revitalization strategy (31), and a systematic safety protection network that considers vulnerable populations should be established to help achieve the goal of the Outline of China National Program for Child Development (2021–2030): child injury mortality should be reduced by 20% compared with 2020 baseline levels (32).

This study has several limitations. First, as a qualitative study, its conclusions primarily rely on participants’ subjective accounts and researchers’ interpretative analysis, potentially introducing interpretative bias. Future studies should incorporate quantitative data to strengthen the argumentative validity. Second, the study sample only covers select regions in Guizhou Province, and the generalizability of findings may be limited by regional cultural and socioeconomic factors. Future research should extend to other regions for comparative analysis.

## 5 Conclusion

This study, based on a social-ecological model, identifies multiple vulnerability factors for unintentional injuries among children aged 3–12 in Guizhou Province. The study discovered that the risk of child injury derives from the interaction across individual, family, school, and community layers. For the first time, the study identifies the cultural phenomenon of “filial silence” and clarifies the influence of filial piety ethics on injury monitoring. It also reveals the triple dilemmas of physical, environmental, and social support faced by children with disabilities, as well as the imbalance in the allocation of safety resources embodied in urban and rural environmental risks. These findings not only challenge health behavior theory via the “cognitive-behavior paradox” but also provide new perspectives for child protection research in developing countries.

## Data Availability

The original contributions presented in the study are included in the article/Supplementary material, further inquiries can be directed to the corresponding author.

## Author’s note

All authors confirm that the following manuscript is a transparent and honest account of the reported research. This research is related to a previous study by the same authors titled “A qualitative study on the current status and needs of unintentional injury prevention and control interventions for children aged 3-12 in Guizhou Province, China” (Frontiers in Public Health, 2025, 13:1712091, doi: 10.3389/fpubh.2025.1712091). Both studies used the same study population (children aged 3–12 years and their caregivers, teachers, and social workers in Guizhou Province) but were designed as two independent sub-studies with separate interview guides, independent data collection, and distinct research objectives. The previous study focused on describing the current status and needs of unintentional injury prevention and control interventions. The current submission focuses on identifying multi-level vulnerability factors (individual, family, school, and community) for unintentional injuries based on the Social-Ecological Model. The two studies collected data independently using different interview guides. Therefore, the current manuscript is not a secondary analysis of the previous dataset. The findings reported here—including the phenomenon of “filial silence,” the triple dilemmas of children with disabilities, and the cognitive-behavior paradox—are novel and were not addressed in the previous publication.
