# Supplementary material for: A qualitative study on the vulnerability factors of unintentional injuries among children aged 3–12 in Guizhou Province, China, based on the social-ecological model
Source: Front Public Health. 2026 Jul 27;14:1853061. doi: 10.3389/fpubh.2026.1853061 (PMC13453836; doi:10.3389/fpubh.2026.1853061)
Supplement: Supplementary file 1 [file Table_1.DOCX]

****Interview Guide****

A Qualitative Study on the Vulnerability Factors of Unintentional Injuries Among Children Aged 3-12 in Guizhou Province, China, Based on the Social-Ecological Model

**Section 1: Focus Group Interview**

Interview Participants: Children aged 3 to 12 (children who are able to clearly express their thoughts and feelings, in groups of 5 to 6).

**1. Description of Unintentional Injury Incident**

- Do you recognize any of these injuries in the picture? Would you be willing to tell me which types of injuries are depicted? (Look at the pictures and say the types of injuries) **Note: The picture is available at the end of this document.**
- Have you ever had any of the injuries shown in the picture in the past year (12 months)? (This can be more than one injury event.)

**------If you have not experienced any of the injuries depicted in the picture:**

① Have any of your close friends suffered from any of these injuries in the picture? Do you know why they were hurt?

**------If you have experienced any of the injuries depicted in the picture:**

①Which of the injuries pictured have you suffered? Where did the injury happen? Why did you get injured? What did you do after the injury?

② Was someone there for you when you were injured? Did you ask anyone for help? How did he help you?

③ Did you tell your parents about the injury? Why? What did they do? (Follow-up questions, as appropriate, to explore parents' reactions to children's unintentional injuries.) Who do you tell when parents are not home? Why?

**2. Children's individual vulnerability factors**

- Where do you usually like to play? Who do you play with? What kind of games do you play there? Can you get hurt in these games? What will you do if you get hurt?
- What chores do you help with at home? Do your parents often watch you while you’re doing them? Have you ever been injured accidentally while doing housework? Can you give me an example?
- Do you understand what things in life are unsafe and should not be done? Who taught you this critical safety information?
- What are some of the ways you protect yourself from getting hurt (for example, how to avoid being burnt or falling down)? Who taught you these methods?

**3. Family vulnerability factors**

**Home environment**

- Which areas in your home do you think are dangerous and likely to cause injuries? Why?
- What activities do you usually do in those places? Have you ever gotten hurt there? How did the injury happen?
- Was anyone with you when you got hurt? Did you ask someone for help? How did they help you?

**Home Safety Education**

- Have your parents taught you how to protect yourself from getting hurt? What methods have they taught you? Could you give an example? How did they teach you—was it through verbal reminders, videos, pictures, or other ways?

**Family Communication**

- Do you enjoy playing with your parents? Why or why not?
- If you want to go somewhere to play with your friends but your parents don’t agree, what would you do?
- When you face difficulties or danger, do you tell your parents right away? Why? How do they help you?

**Family Guardianship**

- Do you live with your parents?

a. If yes: Do you usually take the initiative to talk with them? Do you enjoy chatting with them? Why? How long do your conversations usually last?

b. If not, How often do they come back? Do you usually contact them? How often? Do you enjoy talking to them? Do you miss them? Why?

- Who mainly takes care of you on a daily basis?
- Have your parents ever told you not to do dangerous things? What kinds of things are dangerous? Do you listen to them and avoid doing these risky things?
- If you accidentally do something dangerous, how do your parents usually respond?
- Do your parents let you go out to play alone? Why or why not?

**Family Economic Situation**

- When you want something, like a toy, do your parents buy it for you?

—If not: What kinds of things won’t your parents buy for you? Why? What do you do then? (Follow up based on the situation)

- When do you think your family’s financial situation affects your safety? Can you give an example? (For instance, if you want to go swimming but can’t afford a swimming pool, you might go to a reservoir instead.)

**4. School vulnerability factors**

- Which places at school do you think are dangerous and where students are more likely to get hurt? Why? (whether the floor is too slippery, the corners of tables are sharp, or other reasons)
- What activities do you usually do in those places? Have you ever been injured there? How did the injury happen?
- Was anyone with you at the time of the injury? Did you ask someone for help? How did they help you?
- Have your teachers ever taught you how to protect yourself from getting injured? What methods did they teach you? How did they teach these—by verbal reminders, videos, pictures, drills, or other ways?

**5. Community Vulnerability Factors**

- Which places in your community do you think are dangerous and where you are more likely to get hurt? Why?
- What activities do you usually do in those places? Have you ever been injured there? How did the injury happen?
- Was anyone with you when you got hurt? Did you ask someone for help? How did they help you?
- In your community, what safety features or measures have you seen that protect children? Do you think these are effective?
- What unintentional injury awareness activities for children have you participated in in your neighbourhood? Can you give an example? In what ways were the awareness campaigns conducted? (e.g., videos, talks, verbal instructions, drills, etc.)

**6. Closing Remarks**

- Thank you for sharing your stories. Is there anything else you would like to add?

**Section 2: In-Depth Interview**

Interview Participants: Primary carers of children aged 3–12 years

**1. Description of Unintentional Injury Incident**

- Do you recognise any of these injuries in the picture? Would you be willing to tell me which types of injuries are in the pictures? (Look at the pictures and say the types of injuries.)
- Has your child experienced any of these injuries in the past year (12 months)? (This can be more than one injury event.)

**----- If they have not suffered any of the injuries in the pictures**

① Have any children you know suffered any of these injuries? What do you think is the most important reason for these unintentional injuries?

**------ If they have suffered the injuries pictured**

① What types of injuries has your child had? Where did the injury occur?

② What do you think was the main cause of this injury? How did you hear about the injury? After learning about it, what did you do? (Ask further questions as appropriate to explore the parents’ reactions.)

③ What do you think can be done to prevent these injuries? What should be the division of labour and cooperation among family, school and society?

**2. Children's individual vulnerability factors**

- Where does your child usually like to play? Who do they play with? What games do they play there? Are these games likely to cause injuries? If they do get hurt, what do they usually do?
- What chores does your child usually participate in? Do you or other adults usually supervise them while they do these chores? Has your child ever been injured while doing chores? Could you give an example?
- How would you describe your child’s awareness of safety? Why do you say that? Can you give an example?
- Do you think that unintentional injuries in children are related to their personality or behavioural traits? Which traits do you think make children more prone to such injuries?
- Does your child have any physical or psychological conditions? Do you think these increase their risk of getting injured? Why?

**3. Family vulnerability factors**

**Home environment**

- What areas of the home do you think are dangerous and can easily injure a child? Why?
- What activities do they usually do in those places? Have they ever been injured there? How did the injury happen?
- Were you nearby when your child got hurt? Does your child tell you when they are injured? What do you usually do afterwards?

**Home Safety Education**

- What safety knowledge do you usually teach your child? In what way do you usually teach them? (For example, through videos, verbal reminders, etc.)
- Do you feel your own safety knowledge is sufficient? What other aspects of safety knowledge would you like to add?

**Family Communication**

- Does your child like to play with you? Why or why not?
- If your child wants to go somewhere to play with friends but you don’t agree, how does your child usually respond?
- When your child encounters difficulties or danger outside (for example, getting injured at school), do they tell you right away? Why or why not? How do you usually handle the situation?

**Family Guardianship**

- Does your child live with you and your spouse?

a. If yes: Does the child usually initiate your conversation? Does he enjoy talking to you? Why or why not? How long do these conversations usually last?

b. If no: How often do you or your spouse come home to see your child? Does the child usually initiate contact with you? How often? Do they enjoy talking with you? Why or why not? How long do these conversations usually last?

- Who usually takes care of your child?
- Have you ever told your child not to do dangerous things? Can you give me an example? Will they listen to you and not do these dangerous things?
- If your child accidentally does something dangerous, how do you usually respond?
- Do you allow your child to go out and play alone? Why or why not?
- How long have you been taking care of this child? On average, how much time do you spend caring for your child each day? What are the main caregiving tasks you handle?
- What are the main difficulties and challenges you face while taking care of your child? How do you adjust and cope with them?
- Regarding your child’s safety, when do you feel anxious, exhausted, or experience other negative emotions? What do you usually do at those times? How do you regulate these emotions?
- Do you have enough time and energy for caring for your child? If time and energy are not enough, how do you usually cope?
- If you notice your child doing something unsafe, what do you do? Why?

**Family Economic Situation**

- Do you usually fulfil your child’s requests for entertainment or sports activities from a financial standpoint? If you don’t meet these requests, how does your child typically react?
- Do you think that the family's financial situation affects the probability of a child being unintentionally injured? Can you give examples? (For example, if a child wants to go swimming, he/she may choose to swim in dangerous waters such as a reservoir if the family cannot afford the cost of a swimming pool.)

**4. School vulnerability factors**

- Has your child ever been injured at school? What was the reason for the injury? How did you find out about his injury? What did you do afterwards?
- To the best of your knowledge, what has the school done to prevent and control unintentional injuries to children? In what way was this done? How effective do you think it is? What are the areas that need to be improved?

**5. Community Vulnerability Factors**

- Has your child ever been injured in the community? What was the cause of the injury? How did you find out about it, and what did you do afterwards?
- What has your community done to prevent and control unintentional injuries to children? Through what specific activities or formats (such as safety lectures, informational videos, or emergency drills) are these measures carried out? How effective do you think these efforts are, and what areas need improvement?

— If none: What are the channels through which you generally obtain knowledge about unintentional injuries to children?

- If your community offered lectures on preventing unintentional injuries to children, would you be willing to attend? Why or why not?
- In your opinion, which areas of child safety protection in your community currently need the most improvement?
- To your knowledge, what is society’s attitude toward and level of concern about unintentional injuries to children? Why do you think this is? Could you provide an example?

**6. Closing Remarks**

- Is there anything else you would like to add regarding unintentional injuries to children?

**Section 3: In-Depth Interview**

Interview Participants: Teachers (including school administrators) of children aged 3 to 12 years old

**1. Description of Unintentional Injury Incident**

- Do you recognise any of these injuries in the picture? Would you be willing to tell me which types of injuries are in the pictures? (Look at the pictures and say the types of injuries.)
- How many years have you been working as a teacher? Could you share with me some memorable cases of unintentional injuries involving children that you have encountered?
- In your opinion, what are the main causes of these unintentional injuries to children?
- What do you think can be done to prevent these injuries? What should be the division of labour and cooperation among family, school and society?

**2. Children's individual vulnerability factors**

- Based on your experience, which children are more prone to unintentional injuries? What personality traits or behaviours are these injuries usually associated with? Could you explain the reasons behind this?
- For children who have experienced unintentional injuries, how would you assess their safety awareness? Could you provide a specific example? What aspects of safety awareness do you think they need to enhance? How should it be enhanced?
- How many left-behind children and how many children with disabilities are there in your class (or school)?
- In your experience observing and educating left-behind children and children with disabilities, what do you think are the main causes of their injuries? (Please consider factors at the individual, family, school, and social levels.)
- How would you evaluate the current level of awareness and attention in society regarding the prevention of unintentional injuries to children with disabilities (such as neglect, subtle discrimination, insufficient accessibility facilities, etc.)? What do you think are the reasons behind this situation? Could you illustrate with a specific example?

**3. Family vulnerability factors**

- How well do you understand your students’ family situations (such as their parents' work situation, family financial situation, etc.)? In your opinion, which carer-related factors are most likely to contribute to unintentional injuries in children (e.g., the carer's lack of knowledge, energy, ability, etc.)?
- Which factors within the family (e.g., home environment, family safety education, family supervision, family communication, family economic status, etc.) do you think are related to the occurrence of unintentional injuries in children? Why?
- Compared to children who are neither left-behind nor disabled, how do carers typically respond when left-behind or disabled children get injured? What would they do?
- In your opinion, what shortcomings do carers still have in preventing unintentional injuries to children? How should these be improved?

**4. School vulnerability factors**

- What are the main types of unintentional injuries that occur to children at school? What do you think are the causes?
- Do children tell you when they are unintentionally injured at school? What would you do?
- To your knowledge, what measures has the school taken to prevent unintentional injuries to children? Through what specific activities or formats (such as safety lectures, awareness videos, emergency drills, etc.) etc.)are these measures carried out? How effective do you think these measures are, and what areas need improvement?

**5. Community Vulnerability Factors**

- What has your community done to prevent and control unintentional injuries to children? Through what specific activities or formats (such as safety lectures, informational videos, or emergency drills) are these measures carried out? How effective do you think these efforts are, and what areas need improvement?

— If none: What are the channels through which you generally obtain knowledge about unintentional injuries to children?

- If your community offered lectures on preventing unintentional injuries to children, would you be willing to attend? Why or why not?
- In your opinion, which areas of child safety protection in your community currently need the most improvement?
- To your knowledge, what is society’s attitude toward and level of concern about unintentional injuries to children? Why do you think this is? Could you provide an example?

**6. Closing Remarks**

- Is there anything else you would like to add regarding unintentional injuries to children?

**Section 4: In-Depth Interview**

Interview Participants: Social workers (including social work managers)

**1. Description of Unintentional Injury Incident**

- Do you recognise any of these injuries in the picture? Would you be willing to tell me which types of injuries are in the pictures? (Look at the pictures and say the types of injuries.)
- How many years have you been working in the social work profession? Could you share with me some memorable cases of unintentional injuries involving children that you have encountered?
- In your opinion, what are the main causes of these unintentional injuries to children?
- What do you think can be done to prevent these injuries? What should be the division of labour and cooperation among family, school and society?

**2. Children's individual vulnerability factors**

- Based on your experience, which children are more prone to unintentional injuries? What personality traits or behaviours are these injuries usually associated with? Could you explain the reasons behind this?
- For children who have experienced unintentional injuries, how would you assess their safety awareness? Could you provide a specific example? What aspects of safety awareness do you think they need to enhance? How should it be enhanced?
- How many left-behind children and how many children with disabilities are there in the community you serve?
- Based on your observations and work with families of left-behind and disabled children, what do you think are the main causes of their injuries? (You may analyse from individual, family, school, and social perspectives.)
- How would you evaluate the current level of awareness and attention in society regarding the prevention of unintentional injuries to children with disabilities (such as neglect, subtle discrimination, insufficient accessibility facilities, etc.)? What do you think are the reasons behind this situation? Could you illustrate with a specific example?

**3. Family vulnerability factors**

- How well do you understand the family situations in the community you serve (such as their parents' work situation, family financial situation, etc.)? In your opinion, which carer-related factors are most likely to contribute to unintentional injuries in children (e.g., the carer's lack of knowledge, energy, ability, etc.)?
- Which factors within the family (e.g., home environment, family safety education, family supervision, family communication, family economic status, etc.) do you think are related to the occurrence of unintentional injuries in children? Why?
- Compared to children who are neither left-behind nor disabled, how do carers typically respond when left-behind or disabled children get injured? What would they do?
- In your opinion, what shortcomings do carers still have in preventing unintentional injuries to children? How should these be impro**ved?**

**4. School vulnerability factors**

- Which school-related factors do you think are most likely to contribute to unintentional injuries in children, and why?
- To your knowledge, what measures has the school taken to prevent unintentional injuries to children? Through what specific activities or formats (such as safety lectures, awareness videos, emergency drills, etc.) etc.)are these measures carried out? How effective do you think these measures are, and what areas need improvement?

**5. Community Vulnerability Factors**

- What are the main types of unintentional injuries that occur to children in the community you serve? What do you think are the causes of these injuries?
- What has been done to prevent and control unintentional injuries to children in the community you serve? Through what forms are these measures carried out (e.g., safety lectures, promotional videos, or emergency drills)? How often are these measures conducted? What is the coverage of these services? How effective do you think these measures have been? What areas still need improvement?
- What are the main difficulties and challenges encountered by your social organization in carrying out the prevention and control of unintentional injuries to children? What are the specific reasons for these difficulties and challenges? How should they be dealt with?
- In your opinion, what shortcomings still exist in the current prevention and control measures for unintentional injuries in children? How do these affect the effectiveness of prevention efforts? How can these measures be improved?
- How do you evaluate society’s attitude and level of concern regarding unintentional injuries in children? What do you think are the reasons behind this situation? Could you illustrate with a specific example?
- In your view, how should social organizations, government departments, educational institutions, and families clarify their responsibilities and strengthen collaboration in the prevention and control of unintentional injuries in children to effectively reduce their occurrence?

**6. Closing Remarks**

- Is there anything else you would like to add regarding unintentional injuries to children?

Cartoon Illustrations of Unintentional Childhood Injuries

1. Fall-related Injuries

|  |  |
| --- | --- |
|  |  |

2. Burns/scalds

|  |  |
| --- | --- |
|  |  |

3. Sharp Object Injuries

|  |  |
| --- | --- |
|  |  |

4. Poisoning

|  |  |
| --- | --- |
|  |  |

5. Drowning

6. Road Traffic Injuries (RTIs)

|  |  |
| --- | --- |
|  |  |
|  |  |

7. Animal Bites and Scratches

|  |  |
| --- | --- |
|  |  |

8. Other Injury Types

| Blunt Force Injuries | Electrical Injuries |
| --- | --- |
|  |  |
| Airway foreign bodies | Blast Injuries |
|  |  |
